# Accuracy of IgM antibody testing, FQ-PCR and culture in laboratory diagnosis of acute infection by *Mycoplasma pneumoniae* in adults and adolescents with community-acquired pneumonia

**DOI:** 10.1186/1471-2334-13-172

**Published:** 2013-04-11

**Authors:** Jiuxin Qu, Li Gu, Jiang Wu, Jianping Dong, Zenghui Pu, Yan Gao, Ming Hu, Yongxiang Zhang, Feng Gao, Bin Cao, Chen Wang

**Affiliations:** 1 Department of Infectious Diseases and Clinical Microbiology, Beijing Chao-Yang Hospital, Capital Medical University, Beijing Institute of Respiratory Medicine, Beijing 100020, China; 2 Beijing Centers for Diseases Control and Prevention, Beijing 100020, China; 3 Beijing Hai-Dian Hospital, Beijing 100020, China; 4 YanTai Yu Huang-Ding hospital, Beijing 100020, China; 5 Peking University People’s Hospital, Beijing 100020, China; 6 The Lu-He teaching hospital of the Capital Medical University, Beijing 100020, China; 7 Beijing Da-Xing hospital, Beijing 100020, China; 8 WangJing Hospital of China Academy of Chinese Medical Sciences, Beijing 100020, China; 9 Beijing Hospital, Ministry of Health, Beijing Institute of Respiratory Medicine, Beijing 100020, China

**Keywords:** Community-acquired pneumonia, *Mycoplasma pneumoniae*, Diagnosis, Adult

## Abstract

**Background:**

Diagnosis of community-acquired pneumonia (CAP) caused by *Mycoplasma pneumoniae* in adults and adolescents is hampered by a lack of rapid and standardized tests for detection.

**Methods:**

CAP patients from 12 teaching hospitals were prospectively and consecutively recruited. Basic and clinical information, throat swabs and paired sera were collected. *Mycoplasma pneumoniae* was detected by IgG and IgM antibody tests, fluorescence quantitative polymerase chain reaction (FQ-PCR) and culture. A comparative study of the diagnostic values of three methods, including sensitivity, specificity, positive and negative predictive values and positive likelihood ratio (PLR) was conducted. A fourfold or greater increase of IgG antibody titers of paired sera was set as the diagnostic “gold standard”.

**Results:**

One hundred and twenty-five CAP patients (52.8% males, median age 47 years, range 14–85) were enrolled. Twenty-seven (21.6%) patients were diagnosed with acute *Mycoplasma pneumoniae* infections by the “gold standard”. Specificity values of all three methods were around 90%. An increasing trend of sensitivity, positive predictive value and PLR was found, with the lowest in IgM testing (7.4%, 28.6% and 1.45), intermediate in FQ-PCR (40.7%, 50% and 3.63), and highest in culture (55.6%, 75% and 10.9).

**Conclusions:**

In the defined group of patients, there was a good agreement between positive rate of MP cultivation of throat swabs and acute *M. pneumoniae* infection (PLR of 10.9). Since the sensitivity is low in all of the evaluated methods, the logical approach would be to incorporate PCR, culture and serological tests for optimum diagnosis of acute *Mycoplasma pneumoniae* infections in adults and adolescents.

## Background

*Mycoplasma pneumoniae* (*M. pneumoniae,* MP) is a common human respiratory tract pathogen that causes 6–30% of community-acquired pneumonia (CAP) cases in adults all over the world [1], and especially in China, where the incidence is 20–30% [2,3]. Since the clinical and laboratory manifestations do not differentiate between pneumonia caused by typical or atypical pathogens [4,5], and the commonly described β-lactams are not effective because *M. pneumoniae* lacks a cell wall, adequate laboratory diagnosis of *M. pneumoniae* pneumonia is important.

Diagnosis of *M. pneumoniae* infection in routine clinical practice has been based on serology, culture and polymerase chain reaction (PCR). Among these, paired sera showing a fourfold increase in IgG antibody titer has been considered as a more reliable method for the diagnosis of current *M. pneumoniae* infection [6-8]. However, because of difficulty in obtaining convalescent serum and time constraints, IgG antibody titer tests of paired sera are an epidemiological rather than a diagnostic tool. Culture of this organism is slow [9], and the correlation between culture and infection is tenuous because of the asymptomatic infection caused by *M. pneumoniae*[10,11]. The detection of IgM antibody titer in acute stage serum and fluorescence quantitative PCR (FQ-PCR) of throat swabs are faster techniques, but until now, a direct comparison between paired sera results and the above three assays, especially the FQ-PCR kit approved by State Food and Drug Administration of China, has not been made.

This study is based on the Beijing Network for Adult Community-Acquired Pneumonia, which consists of 12 teaching hospitals in Beijing. We evaluated the accuracy of IgM serology, FQ-PCR and culture for early diagnosis of CAP caused by *M. pneumoniae*, with a fourfold or greater increase of IgG antibody titers of paired sera as the “gold standard”.

## Methods

### Study population

A prospective study was conducted in 12 teaching hospitals (Beijing Chao-Yang Hospital, Beijing Haidian Hospital, YanTai Yu Huangding Hospital, Peking University People’s Hospital, Luhe Teaching Hospital of the Capital Medical University, WangJing Hospital of China Academy of Chinese Medical Sciences, Peking Union Medical College Hospital, China-Japan Friendship Hospital, Beijing Friendship Hospital, Beijing Pinggu Hospital, Huairou the First Hospital and Air Force General Hospital, PLA) in Beijing, China. From October 2010 to September 2011, adults and adolescent patients (≥ 14 years of age) with radiographically confirmed CAP were enrolled consecutively. *M. pneumoniae* was detected by the central laboratory, Clinical Microbiological Laboratory of Beijing Chao-Yang Hospital. Patients with immunosuppression and those who had received immunosuppressive therapy were excluded. In addition, pregnant or lactating mothers, patients from nursing homes, patients whose onset time was longer than 7 days or patients who had been admitted to a hospital longer than 2 days within the last 90 days were also excluded. Clinical features, including comorbidities (such as diabetes, heart diseases, cerebral vascular disease, chronic lung and renal disease) and laboratory data, were recorded on a data sheet and then entered into a computer database when patients were enrolled. The study was approved by the institutional review board in Beijing Chao-Yang Hospital. Written informed consent was provided by all adults and the parents of patients aged less than 18 years.

### Microbiological laboratory tests

Throat swabs and first serum samples were obtained on admission, and convalescent serum samples were obtained 2–4 weeks later. Throat swabs were performed with 2 ml transport broth medium (CM403, OXOID). Specimens were stored at −80°C until transportation on ice to the laboratory within 2 weeks.

For *M. pneumoniae* culture, throat swab specimens were vortexed, supplemented with amphotericin B and penicillin, and inoculated into SP-4 medium. The medium was incubated at 37°C, and observed daily for 2–6 weeks for a decrease in pH (a red to yellow color change). Positive cultures were confirmed by PCR assay as previously reported [12].

DNA was extracted from 200 μl of throat swab sample by manual nucleic acid extraction (Qiagen QIAmp DNA Mini Kit, Valencia, CA). *M. pneumoniae* was detected by a commercial FQ-PCR kit (Daan Gene, Guangzhou, China) approved by State Food and Drug Administration, targeting the 16S ribosomal RNA gene [GenBank:AF132740]. The FQ-PCR mixture was prepared in a total volume of 45 μl, containing 3 μl of sample DNA. FQ-PCR was performed under the following conditions: initial activation at 93°C for 2 min, followed by 10 cycles at 93°C for 45 s and 55°C for 1 min, and 30 cycles at 93°C for 30 s and 45°C for 30 s. The results were displayed as qualitative. An internal control, targeted human ribonuclease protein (hRNP) gene, was incorporated. The amplifications were performed using the AB 7500 Real Time PCR System (Applied Biosystems, Foster City, CA) according to the manufacturer’s instructions.

The specimens were quantitatively tested for IgG and IgM antibodies against *M. pneumoniae* using the Virion/Serion ELISA kit (GmbH Germany ESR127G and ESR127M). Antibody activities were given in U/ml. Based on the manufacturer’s recommendation, MP IgG calculation was interpreted as follows: positive results (> 30 U/ml), borderline results (20–30 U/ml) and negative results (< 20 U/ml); and for IgM: positive results (> 17 U/ml), borderline results (13–17 U/ml), and negative results (< 13 U/ml). All ELISA reactions were performed according to the manufacturer’s instructions on the Thermo Multiskan MK3 (Thermo Fisher Scientific, Waltham, MA) system by the same technicians.

Microbiological examination was also performed in throat swab, sputum, urine and blood. The etiology was considered definite if one of the following criteria was met: (1) positive bacterial culture; (2) positive urinary antigen for *L pneumophila* (Binax Now *L pneumophila* urinary antigen test; Trinity Biotech, Bray, Ireland); (3) positive urinary antigen for *S pneumoniae* (Binax Now *S pneumonia* urinary antigen test; Emergo Europe, The Netherlands); (4) detection of respiratory viruses by RT-PCR using a Seeplex RV Detection Kit (Seegene Biotechnology Inc., Seoul, Korea), including respiratory syncytial virus A and B, influenza virus (IFV) A and B, parainfluenza virus (PIV) 1–4, human rhinovirus (HRV), enterovirus, human coronavirus (229E/NL63 and OC43/HKU1), human metapneumovirus (hMPV), adenovirus (AdV), and human bocavirus.

### Statistical analysis

Comparisons of clinical characteristics and clinical outcomes were conducted between patients with *M. pneumoniae* positive and negative group, using an unpaired Student’s *t*-test, the Mann–Whitney test, or the Chi-square test (SPSS for Windows 16.0). A p-value < 0.05 was considered statistically significant. Sensitivity, specificity and predictive values were determined by standard procedures. Clinical diagnostic values of different tests were evaluated by likelihood ratios. Strong evidence to rule in diagnoses in most circumstances when the positive likelihood ratio was above 10 [13].

## Results

During our 2-year prospective study, 125 CAP patients with paired sera meeting the inclusion criteria were enrolled. There were 66 males (52.8%) and 59 females (47.2%), and the patients’ ages ranged from 14 to 85 years (median 47 years) (see Table 1). Overall, acute *M. pneumoniae* infection was diagnosed in 27/125 (21.6%) patients showing a fourfold or greater increase in IgG antibody titer of paired sera. Twenty-three patients had causative pathogens other than *M. pneumoniae*. Among these patients, one was positive for PIV types 1 and 2, and IFVA; one was positive for IFVA and *Pseudomonas aeruginosa*; one was positive for IFVA and *Klebsiella pneumoniae*; nine were positive for IFVA; three were positive for adenovirus; three were positive for PIV type 1; one was positive for hMPV; one was positive for HRV; one was positive for PIV type 2; and one was positive for PIV type 3. Patients positive for *M. pneumoniae* had a median age of 34 years (range 16–77 years), which was significantly younger than *M. pneumoniae*-negative patients (54.5; range 14–85) years, p = 0.002). *M. pneumoniae* infections were less likely to have comorbidities (p = 0.039).

**Table 1:** Demographic and Clinical features and laboratory findings of studied patients

	**All patients**	**MP positive**	**MP negative**	***P *value**
	**N = 125 (%)**	**N = 27 (%)**	**N = 98 (%)**	
**Age (years)**	47 (14–85)	34 (16–77)	54.5 (14–85)	0.002
**Gender (male)**	66 (52.8)	13 (48.15)	53 (54.08)	0.665
**Comorbidities**	30 (24.0)	2 (7.41)	28 (28.57)	0.039
**Current smoker**	38 (30.4)	6 (22.22)	32 (32.65)	0.57
**Symptoms**				
Fever	110 (88.0)	25 (92.59)	85 (86.73)	0.521
Duration from onset (days)	3 (2–5)	4 (3–5.5)	3 (2–5)	0.013
T_max_ (°C)	39.06 ± 0.58	39.22 ± 0.57	39 ± 0.58	0.113
Cough	114 (91.2)	27 (100.00)	87 (88.78)	0.119
Sputum production	83 (66.4)	20 (74.07)	60 (61.22)	0.37
Dyspnea	22 (17.6)	7 (25.93)	15 (15.31)	0.253
Chest pain	17 (13.6)	4 (14.81)	13 (13.27)	0.761
Digestive symptoms	11 (8.8)	5 (18.52)	6 (6.12)	0.052
Heart rate/min	85.36 ± 18.07	96.7 ± 16.71	85.16 ± 11.27	0.000
Respiratory rate/min	22.32 ± 12.38	20 ± 1.78	20.13 ± 2.86	0.819
**Laboratory findings**				
White blood cell (10^9^/L)	8.78 ± 4.16	8.16 ± 3.11	8.94 ± 4.4	0.386
Neutrophil (%)	71.73 ± 12.10	71.11 ± 9.3	71.89 ± 12.8	0.77
Lymphocyte (%)	19.39 ± 9.99	18.5 ± 7.14	19.6 ± 10.64	0.614
Hematocrit (%)	42.15 ± 28.86	39.86 ± 4.7	42.7 ± 32.29	0.659
Hemoglobin (g/L)	132.54 ± 20.72	135.68 ± 16.05	131.71 ± 21.78	0.387
Platelet (10^9^/L)	207.77 ± 68.55	192.77 ± 50.94	211.76 ± 72.21	0.211
ESR (mm/hr) n = 85	41.51 ± 26.14	40.05 ± 26.19	41.92 ± 26.31	0.785
C-reactive protein (mg/L) n = 83	66.78 ± 65.65	63.75 ± 51.97	67.74 ± 69.78	0.841
**Antibiotic usage before admission within 1 week**	77 (61.6)	18 (66.67)	59 (60.2)	0.657
**Site of care (n = 125)**				0.667
Outpatient	51 (40.8)	10 (37.04)	41 (41.84)	
Hospitalization	74 (59.2)	17 (62.96)	57 (58.16)	
**Duration of hospitalization (days)**	14 (10–18)	13 (6.5-19)	14 (11–18)	0.549
**Days after onset at the IgM testing**	4 (1–7)	4 (2–7)	3.5 (1–7)	0.246

Note: The data are presented as means ± standard deviations, no./total no. (%), or median (range).

Comparison is conducted between MP positive (n = 27) and negative (n = 98) group.

There was no difference in smoking status between the two groups. A similar distribution of most symptoms were found in both groups on admission, with the exception that the duration of fever before admission was longer in the group with *M. pneumoniae* infection than in the group without (p = 0.013). The group with *M. pneumoniae* infection had a higher mean heart rate (p < 0.001). The two groups had a similar pattern of biochemical and haematological findings. There was no difference in use of antibiotics, treatment status or days after onset of the IgM testing between the two groups.

The results of IgM testing, FQ-PCR and culture were compared (see Table 2). *M. pneumoniae* positive rates evaluated by IgM antibody testing in acute stage serum (5.6%), FQ-PCR (17.6%) and culture (16%) were lower than the result of paired sera (21.6%). Among the 27 patients positive for *M. pneumoniae*, only two (7.4%) were found to be positive by IgM antibody testing, 11 by FQ-PCR (40.7%) and 15 by culture (55.6%). For all of the 103 non-*M. pneumoniae* infections tested by FQ-PCR, hRNP gene had been successfully amplified (data not shown). Among the discordant results, eight patients showed a fourfold or greater increase in IgG titers, but had negative results in all three methods. Fifteen patients were negative by paired sera, while positive by other methods. Among them, four were positive by both FQ-PCR and culture, one was positive by FQ-PCR and IgM test, one was positive by culture and IgM test, six were positive by FQ-PCR, and three were positive by IgM test. When evaluated by the Japanese Respiratory Society (JRS) scoring system [14], 17 patients positive by paired sera and 40 patients negative by paired sera scored ≥ 4.

**Table 2:** ***M. pneumoniae *detection by IgM antibody testing of acute serum, FQ-PCR, culture and IgG titer of paired sera in adults and adolescents with CAP in Beijing**

		**IgG antibody titers of paired sera with a fourfold or greater increase (number (%))**		
		**Positive**	**Negative**	**Total**
IgM result	Positive	2 (1.6)	5 (4.0)	7 (5.6)
	Negative	25 (20.0)	93 (74.4)	118 (94.4)
FQ-PCR result	Positive	11 (8.8)	11 (8.8)	22 (17.6)
	Negative	16 (12.8)	87 (69.6)	103 (82.4)
Culture result	Positive	15 (12.0)	5 (4.0)	20 (16.0)
	Negative	12 (9.6)	93 (74.4)	105 (84.0)
Total		27 (21.6)	98 (78.4)	125

By using paired sera showing a fourfold or greater increase in IgG antibody titer as the “gold standard”, diagnostic parameters of the three methods have been evaluated (see Table 3), including sensitivity, specificity, positive and negative predictive values (PPV and NPV respectively) and positive likelihood ratio (PLR). Specificity and NPV of all three methods were high, around 90% in specificity and around 80% in NPV. A similarly increasing trend of sensitivity, PPV and PLR was lowest in IgM antibody testing (7.4%, 28.6% and 1.45, respectively), intermediate in FQ-PCR (40.7%, 50% and 3.63, respectively), and highest in culture (55.6%, 75% and 10.9, respectively).

**Table 3:** Diagnostic values of laboratory test methods with a fourfold or greater increase of IgG antibody titers of paired sera as the “gold standard”

	**Sensitivity (%)**	**Specificity (%)**	**PPV (%)**	**NPV (%)**	**PLR**
IgM result	7.4	94.9	28.6	78.8	1.45
FQ-PCR result	40.7	88.8	50	84.5	3.63
Culture result	55.6	94.9	75	88.6	10.9

PPV: Positive predictive values; NPV: Negative predictive values; PLR: Positive likelihood ratio.

## Discussion

Our results show that *M. pneumoniae* is an important pathogen of adolescent and adult CAP patients, consistent with the findings of the CAPNETZ report in 2009 [15]. In the present study, we also demonstrate that, compared with non-mycoplasma infections, CAP patients infected with *M. pneumoniae* are younger, less likely to have comorbidities, and have longer duration of fever before admission.

After infection by *M. pneumoniae*, IgM antibodies appear during the first week of the illness, and reach peak titers during the third week. Martínez et al. have found that the sensitivity of IgM assay was 33.3% in diagnosis of acute *M. pneumoniae* infection in adult CAP patients [16]. In our study, however, the sensitivity of the IgM assay is only 7.4% compared with a fourfold increase of IgG titers. In some adult patients, the antibodies are constantly negative or produced 15 days after onset as a result of multiple previous infections [17,18]. Therefore, using the IgM assay as the sole test may provide inadequate information.

When compared with PCR, the sensitivity of culture from respiratory specimens has turned out to be as low as 61.5% [19]. Similarly, She et al. reported that culture was unacceptably insensitive for diagnosing *M. pneumoniae* infection [7]. In our study, however, there was no difference between the positive rates of the two methods, just as Waris et al. found in children [20]. Furthermore, the values of diagnostic parameters, including specificity, sensitivity, positive and negative predictive values and positive likelihood ratio, are the highest among the three methods evaluated here. In particular, a PLR of 10.9 means that there is a reasonable correlation between culture results and true infection. However, the prolonged turnaround time limits the clinical application of culture.

Compared with serology and culture, PCR (especially real-time PCR) is more rapid, practicable and sensitive, and has been suggested to be more suitable for early diagnosis of acute *M. pneumoniae* infection [21,22]. Touati et al. have evaluated five commercial kits based on PCR, and found that the sensitivities of the kits were 62–98% [23]. Similarly, Martínez et al. observed the sensitivity, specificity, PPV and NPV of the PCR to be 66.7%, 98.5%, 78.3% and 97.3%, respectively [16]. In the present study, however, the sensitivity, specificity, PPV and NPV of the FQ-PCR kit targeted to 16 S rRNA gene were 40.7%, 88.9%, 50% and 84.6%, respectively. The low sensitivity of PCR testing may be due to the low bacterial load resulting from previous antimicrobial treatment or dilution of the sample in the throat swabs below the limit of detection. The detection limit of our FQ-PCR kit is stated as 10 copies per μl in the manual, which is lower than 0.1–1 copies per μl that other commercial kits reported [23,24]. In addition, it was found that the efficiency of the PCR assay could be influenced by sample type, and sputum could be superior to other respiratory samples including throat swab [25].

Possible reasons contributing to discordant results between FQ-PCR or culture, and IgG serology have been explored. Four patients (one positive by FQ-PCR and culture, and three positive by FQ-PCR) might represent asymptomatic carriage of *M. pneumoniae* as a result of persistence from a previous infection, since these patients’ respective CAP etiologies had been identified with IFVA, hMPV, AdV and HRV. Five patients (three positive by FQ-PCR and culture, and two positive by FQ-PCR or culture and IgM test) showed 2.5–3.8 fold increase of IgG response, which could be explained by an inadequate time interval (mean interval was 17 days). The remaining three patients (only positive by FQ-PCR) were 69, 73 and 74 years old. They failed to develop IgG antibody response in paired sera, which could be interpreted as a deterioration of the immune response due to ageing [26]. These cases highlight the limitations of a serological diagnosis, which could be affected by the timing of specimen collection and the age of the patient.

Recently, in China, Yin et al. demonstrated the good sensitivity and specificity of the JRS scoring system for the early presumptive diagnosis of *M. pneumoniae* pneumonia [14]. In our cohort, we showed that with a fourfold or greater increase of IgG antibody titers of paired sera as the “gold standard”, the sensitivity and specificity of JRS scoring were 63% and 59.2%, respectively. With positivity either by PCR, culture, IgM, or a fourfold increase of IgG as the diagnosis for *M. pneumoniae* pneumonia, the sensitivity decreased to 61.9% and specificity increased to 74.7%.

The limitations of the study included: 1) because of cost consideration, we evaluated only one FQ-PCR kit approved by State Food and Drug Administration of China; and 2) in patients who had received antibiotics, including macrolides and fluoroquinolones, the positive rates of culture and FQ-PCR may have been decreased.

## Conclusions

In the defined group of patients, there was a good agreement between positive rate of MP cultivation of throat swabs and acute *M. pneumoniae* infection (PLR of 10.9). Since the sensitivity was low in all evaluated methods, the logical approach would be to incorporate PCR, culture and serological tests for optimum diagnosis of acute *M. pneumoniae* infections in adults and adolescents.

### Ethical approval

The study was approved by the Beijing Chao-yang Hospital Ethics Committee and written informed consent was provided by all adults and the parents of patients aged less than 18 years.

## Competing interests

The authors declare that they have no competing interests.

## Authors’ contributions

QJX carried out all laboratory tests, performed the statistical analysis, participated in the design and coordination of the study and drafted the manuscript. GL, WJ, DJP, PZH, GY, HM, ZYX and GF participated in enrollment of CAP patients and recorded the demographic information. CB and WC participated in the design and coordination of the study, and helped to draft the manuscript. All authors read and approved the final manuscript.

## Pre-publication history

The pre-publication history for this paper can be accessed here:

http://www.biomedcentral.com/1471-2334/13/172/prepub
